# Reconstructing the plinian and co-ignimbrite sources of large volcanic eruptions: A novel approach for the Campanian Ignimbrite

**DOI:** 10.1038/srep21220

**Published:** 2016-02-17

**Authors:** Alejandro Marti, Arnau Folch, Antonio Costa, Samantha Engwell

**Affiliations:** 1 Barcelona Supercomputing Center (BSC-CNS), Barcelona, Spain; 2 Istituto Nazionale di Geofisica e Vulcanologia, Sezione di Bologna, Italy; 3 Istituto Nazionale di Geofisica e Vulcanologia, Sezione di Pisa, Italy

## Abstract

The 39 ka Campanian Ignimbrite (CI) super-eruption was the largest volcanic eruption of the past 200 ka in Europe. Tephra deposits indicate two distinct plume forming phases, Plinian and co-ignimbrite, characteristic of many caldera-forming eruptions. Previous numerical studies have characterized the eruption as a single-phase event, potentially leading to inaccurate assessment of eruption dynamics. To reconstruct the volume, intensity, and duration of the tephra dispersal, we applied a computational inversion method that explicitly accounts for the Plinian and co-ignimbrite phases and for gravitational spreading of the umbrella cloud. To verify the consistency of our results, we performed an additional single-phase inversion using an independent thickness dataset. Our better-fitting two-phase model suggests a higher mass eruption rate than previous studies, and estimates that 3/4 of the total fallout volume is co-ignimbrite in origin. Gravitational spreading of the umbrella cloud dominates tephra transport only within the first hundred kilometres due to strong stratospheric winds in our best-fit wind model. Finally, tephra fallout impacts would have interrupted the westward migration of modern hominid groups in Europe, possibly supporting the hypothesis of prolonged Neanderthal survival in South-Western Europe during the Middle to Upper Palaeolithic transition.

Volcanic super-eruptions, those that eject magma in excess of 450 km^3^ Dense Rock Equivalent (DRE) or ~1,000 km^3^ of volcanic ash deposits^1^,^2^, may have catastrophic long-term global impacts. Despite the low probability of occurrence in relation to human life-spans, the probability increases significantly when we consider the time-scales of civilizations^1^. Many aspects of volcanic super-eruptions are not well understood due to a lack of historical precedents, and such eruptions must be reconstructed from their geological deposits^2^. Reconstructing the volume and tephra dispersal from volcanic super-eruptions is necessary to gain further insight into these catastrophic events and assess their widespread impact on humans, ecosystems and climate. Recent studies^3^,^4^,^5^ have demonstrated that the capability of numerical models^6^ to reconstruct tephra dispersal from these events has greatly improved in recent years.

Commonly associated with caldera-forming events, super-eruptions often include multiple eruptive sources with different styles of ash injection^7^. A common scenario begins with Plinian column destabilization and/or structural collapse of a caldera to produce a collapsing fountain that sheds pyroclastic flows. These high-density flows spread laterally along the ground at high-speeds^8^, eventually leading to formation of secondary, co-ignimbrite plumes^9^ (Fig. 1). Source conditions for co-ignimbrite plumes vary considerably from those of Plinian, with much larger source radii, lower initial ascent velocities and finer granulometry. However, previous numerical studies have simplified the characterization of volcanic super-eruptions to a single eruptive source, potentially leading to inaccurate estimations of their eruption dynamics. In order to evaluate the magnitude of each eruptive phase, it is critical to constrain their eruption dynamics and quantify optimal eruption source parameters (ESPs; i.e. erupted mass, mass flow rate, eruption duration, plume height and total grain size distribution) that best represent each phase of the eruption.

In eruptions where both Plinian and co-ignimbrite sources (referred to as phases when separated in time during the eruption) have occurred, tephra deposits commonly have bimodal grain size distributions at individual sites^10^. A number of processes have been invoked to explain this bimodality, typically ascribed to depositional processes such as aggregation^11^. However, such bimodality has also been interpreted as representing different eruptive phases, specifically Plinian versus co-ignimbrite^10^,^12^,^13^,^14^. Inaccurate assessment of the proportion of co-ignimbrite ash in the deposit in distal areas can lead to an overestimation of the volume of the Plinian deposit^15^.

Atmospheric transport of tephra released during this type of eruption is driven by the interaction of the volcanic plume and the atmospheric wind field^16^. Plumes from high-intensity eruptions can be injected high into the stratosphere, reaching a maximum column height and intruding laterally at neutral buoyancy level (NBL) as a gravity current (Fig. 1). This current can spread at velocities exceeding those of the surrounding winds, affecting tephra transport and deposition near the source^16^,^17^. As particles are deposited and air is entrained, the plume density decreases and momentum reduces such that, at a certain distance, atmospheric turbulence and wind advection become the dominant atmospheric transport mechanisms^17^. Neglecting the gravitational spreading of the umbrella cloud in tephra dispersal simulations could misrepresent the interaction of the volcanic plume and the atmospheric wind field, especially for high-intensity eruptions and for proximal deposition of tephra^5^.

The trachytic-phonolitic Campanian Ignimbrite eruption^18^, the largest eruption of the last 200 ka in Europe, erupted from the Phlegrean Fields on the Bay of Naples (Italy) ~39,300 years ago^19^. Geological evidence suggests that the eruption had two main phases; beginning with a sustained Plinian phase^20^ followed by a secondary co-ignimbrite phase^15^,^21^. The upper portions of the Plinian deposits contain evidence for initiation of column collapse (generating a crater 13 km in diameter^18^) and are overlain by massive ignimbrite deposits^20^, with local thicknesses exceeding 100 m. These deposits were emplaced by pyroclastic density currents that travelled in excess of 80 km from source^22^, and resulted in formation of the co-ignimbrite plume(s)^15^. While the dynamics and physical characteristics of proximal deposits have been widely discussed in the literature^13^,^18^,^23^,^24^, dispersal and volume estimates of the associated distal fallout deposit are still poorly constrained^21^, despite containing a significant portion of the erupted material.

According to recent studies, the resulting stratospheric aerosol cloud would have induced a “volcanic winter”^25^ with a cooling effect of ~6–9 °C in Eastern Europe^26^. Additionally, it has been debated that the eruption, boosted by the impact of the broadly synchronous Heinrich Event 4^27^, contributed to the Middle to Upper Palaeolithic transition^22^,^28^.

In a recent work, Costa *et al.* (2012) combined FALL3D ash dispersion model simulations, analysis of an ensemble of wind fields, and thickness measurements of the tephra deposit to quantify volcanic ash dispersal of the CI eruption. The dispersal model was used in conjunction with a downhill simplex inversion method (DSM)^3^,^4^,^29^ to investigate eruption dynamics. However, Costa *et al.* (2012) reconstructed the eruption as a single-phase event and neglected the gravitational spreading of the umbrella cloud in their tephra dispersal simulations, overlooking potentially important eruption processes. Reconstruction of the eruption as a two-phase event can provide a more realistic characterization of the eruption and allow for a better estimation of its duration.

Here, we develop on the Costa *et al.* (2012) computational approach to 1) reconstruct, for the first time, the duration and contribution of the two phases of the CI super-eruption and, 2) evaluate the effect of gravitational spreading of the umbrella cloud by coupling FALL3D with a model that accounts for the gravity-driven transport in the umbrella cloud^16^. To achieve these objectives, we performed a two-phase inversion on dataset 1, containing 10 deposit thickness measurements with distinct bimodality from which a Total Grain Size Distribution (TGSD) was reconstructed (Table 1). This is a more complex distribution than that used by Costa *et al.* (2012), who assumed an empirically parameterized TGSD bi-log-normal distribution. Results from our two-phase inversion were validated against an independent dataset, dataset 2, consisting of 114 unimodal observations spanning across the dispersal area (see Methods for more details). Figure 2 shows the sampling locations for each dataset along with their reconstructed Total Grain Size Distribution (TGSD). To verify the consistency of the combined two-phase inversion results, we performed an additional single-phase inversion using dataset 2. Finally, we discuss the environmental and climate-forcing implications associated with the eruption to provide insight into its contribution to the Middle to Upper Palaeolithic transition.

## Results

### Modelling the CI eruption as a two-phase event

We performed a model inversion to infer the ESPs (see Table 2) that best represent the tephra deposits for each eruptive phase (dataset 1). A combined forward simulation using best-fit parameters was used to reconstruct the full CI event. Simulation results, presented in Table 2, are validated against tephra thicknesses across the dispersal area (dataset 2).

### Plinian phase

Best-fit results from the inversion model indicated that the eruption began with a short (4 h), high-intensity Plinian explosive phase that produced a column 44 km in height, and a mass eruption rate (MER) of 3.75 × 10^9^ kg/s. Results also showed the vertical mass distribution can be characterized by a value of the Suzuki coefficient^30^ commonly assumed for Plinian eruptive columns (*A* = 4). The Plinian phase deposited a total volume of 54 km^3^ of tephra (~23 km^3^ DRE), accounting for 26% of the total fallout deposit volume, and covering an area of ~1.3 million km^2^ with deposits greater than 0.5 cm in thickness. Figure 3a shows that Plinian lapilli and coarse ash were predominantly deposited in southern Italy with deposit thickness decreasing with distance in concordance with data reported in dataset 1. The correlation coefficient between the observed thicknesses in dataset 1 and the modelling results was 0.76 (Fig. 3e), with a relative root mean square error (RMSE) of 0.10.

### Co-ignimbrite phase

Best-fit results from this phase suggested the co-ignimbrite column(s) reached 37 km in height (with a Suzuki coefficient of *A* = 9), fed by an average MER of ~2.3 × 10^9^ kg/s over approximately 19 h and produced deposits ~154 km^3^ (~62 km^3^ DRE) in volume. The fallout from the co-ignimbrite phase (Fig. 3b), much richer in fines than the Plinian phase, was spread over an area of ~3 million km^2^ (thickness ≥ 0.5 cm), and would have represented almost 74% of the total bulk volume for the eruption. These results confirm the key role of the co-ignimbrite fallout in the total bulk volume^13^,^15^,^21^ and is consistent with fluid dynamics models indicating that co-ignimbrite plumes from very high-intensity eruptions (~10^9^ kg/s) distribute a major proportion of fine grained particles into the stratosphere^31^. Assuming that 35-45% of the erupted material was elutriated from the pyroclastic density current^32^, the total MER for the ignimbrite phase would have been considerably higher than our estimates (up to ~5–6 × 10^9^ kg/s). The correlation coefficient between observations (dataset 1) and simulation results was 0.83 (Fig. 3e), with a relative RMSE of 0.30.

### Combined phases

Using the optimal ESPs resulting from the Plinian and co-ignimbrite phase inversions, we simulated the dispersal from the CI eruption (Fig. 3c) obtaining an eruption duration of 23 h and a total deposit volume of ~208 km^3^ (~84 km^3^ DRE). Tephra fallout would have covered an area of over ~3 million km^2^ (thickness ≥ 0.5 cm). Given volume estimates of 180 to 380 km^3^ for the proximal pyroclastic density current deposits^21^, the total bulk volume would range from 388 to 588 km^3^ (155–235 km^3^ DRE). We used dataset 2 to validate results from the combined phases, obtaining a correlation coefficient of 0.81, a RMSE of 0.18 and a bias of 0.21.

### Modelling the CI eruption as a single-phase event

For the purpose of comparison, we performed an additional single-phase inversion using dataset 2. Best-fit results from (Table 2) suggested a column height of 38 km (with a Suzuki coefficient of *A* = 9), an average MER of ~2.6 × 10^9^ kg/s and a duration of 23 hours. The tephra volume deposited (Fig. 3d) would have totalled ~211 km^3^ (~84 km^3^ DRE). This volume is consistent with the ~208 km^3^ obtained by the two-phase combined inversion. The correlation coefficient between the observed and the simulated thickness is 0.79, with a relative RMSE of 0.27 and bias of 0.47.

### Atmospheric umbrella cloud spreading

We find the gravity-driven transport to be dominant for the first hour of the eruption with an effective radial velocity of ~130 m/s, resulting in an umbrella cloud radius of <100 km. Model results show the effect of atmospheric gravity-driven transport to be significant in proximal areas, increasing tephra deposition by 1.5–2 times NE from the source and decreasing up to 50% in the eastern Mediterranean region (online supplemental material S1). The gravity-current model predicts a fully passive regime^16^ after ~3 hours, with a corresponding umbrella cloud radius of ~430 km. At this distance, atmospheric turbulence and wind advection would have dominated tephra transport for the CI eruption.

## Discussion

We uncover valuable new results and present new methods for reconstruction of the volume and tephra dispersal of the 39 ka CI super-eruption. Our computational approach infers ESP values for both phases, Plinian and co-ignimbrite, of the eruption accounting for the gravitational spreading of the umbrella cloud. This novel approach improves modelled tephra distribution across the dispersal area, and reduces the RMSE of the single-phase inversion by ~33% (0.18/0.27) and bias by ~52% (0.21/0.47). Total volumes, durations, phase-averaged MER, column heights, and mass distributions (Suzuki coefficient *A*) for both (single and two-phase) inversions are consistent (i.e. total tephra volume differed by less than 1.5%), suggesting that results from the two-phase reconstruction are robust.

We also compare the results from our combined two-phase simulation with those reported in the Costa *et al.* (2012) best fit single-phase simulation^3^ (correlations coefficients of 0.81 and 0.77, respectively). Our best-fit simulation uses a higher MER (75% increase), lower total volume (15% decrease) and shorter duration (5 times) of the climatic phase of the eruption using dynamics that are more consistent with geological interpretations of the event. In addition, our two-phase reconstruction is in better agreement with the Koyaguchi *et al.*’s collapse conditions (see Fig. 6 in their study)^33^, and the Woods and Wohletz column-height/MER relationship (see Fig. 3 in their study)^9^. Concerning the reconstruction of the deposit, our simulations suggest a slightly thinner tephra blanket in the East Black Sea than Costa *et al.* (2012). This is mainly due to the different TGSDs used in the two studies (the TGSD used here is reconstructed from field data limited to deposits within 900 km from source and is fines depleted with respect to Costa *et al.* 2012).

In general terms, our proximal tephra fallout is consistent with previous studies^15^,^24^. Much of the dispersal area was covered by 1–10 cm of ash, including regions from the Mediterranean and Ionian Sea to the east European Plains. Dispersal results also predict a thick tephra deposit (10–20 cm) covering regions of present-day Macedonia, Bulgaria and Romania, which is consistent with tephra deposits in the south-eastern Romanian loess steppe^34^. Fine ash aggregation processes could explain this secondary maximum thickness. In order to account for ash aggregation we use the model of Cornell *et al.* (1983)^35^, who analysed the CI deposit (Y5 ash layer) and determined that 50, 75, and 100% of the 63–44, 44–31, and <31 μm ash could be considered as a single aggregated class. The model assumes a simplified distribution of aggregates in the eruption column with a single effective diameter and constant density. More sophisticated aggregation models could not be employed as they are too computationally intensive for an inversion analysis. Ultra-distal dispersal from our simulations (>2500 km NE from source) is consistent with analyses of the CI ash layer identified in the Russian Plain^21^.

Reconstruction of the Plinian phase indicates that tephra volume from this phase is 2–3 times larger than previous studies^21^. However, the maximum height and mass distribution of the eruptive column in our simulations is consistent with the height estimated by field and laboratory analyses^20^ of a sustained Plinian column with maximum mass distribution at 3/4 of the column height^36^,^37^. The resulting eastern dispersion trend is compatible with proximal Plinian fall products^15^,^20^. On the other hand, tephra fallout from the co-ignimbrite phase is similar to higher-end estimates (73–140 km^3^) from previous studies^21^. The relative proportion of the distal co-ignimbrite tephra (130–900 km from source) over the total bulk volume for the eruption is consistent with estimates reported by Engwell *et al.* (2014)^13^ (74% versus 60 ± 6%). Deposits in Eastern Europe and North Africa in particular, are predominately composed of co-ignimbrite tephra. Figure 4 shows the contribution from the Plinian and co-ignimbrite tephra to the total bulk volume. At distances greater than 900 km from the source, it is not possible to quantitatively determine the relative proportions of tephra from the different phases due to the unimodal nature of the deposits^13^. However, the rapid decrease in the Plinian component between 800 and 900 km indicates that ultra-distal tephra deposits (~1–2 cm) would have been predominantly co-ignimbrite in origin^21^.

The inclusion of the gravity-driven transport improves tephra distribution in proximal areas reducing the overall RMSE by ~20% and the bias by ~20% (0.28 to 0.21). Contrary to other large explosive volcanic eruptions (e.g. Mt. Pinatubo^16^, Toba Tuff^4^), where cloud spreading and spinning velocities exceed typical stratospheric wind speeds^17^, gravity-current transport was dominant for the first hour only, producing an umbrella cloud radius of ~100 km upwind. This result is in agreement with findings of Giaccio *et al.* (2012)^38^, who reported the absence of tephra deposits associated with the CI event at the Sulmona intermountain basin, less than 150 km north of the vent, suggesting CI tephra transport was dominated by wind advection. An explanation for this phenomenon is associated with the inferred strong stratospheric winds (~90 m/s) above the vent, which could have prevailed over the effect of the spreading umbrella cloud (online supplemental material S1). Stratospheric wind speed values above the vent are consistent with the mean of the 10 best meteorological fields found by Costa *et al.* (2012), which ranged from ~55 to ~95 m/s. This could support the idea that during the last Glacial period, winds were stronger than those in present-day^39^. The passive transport dominance during the CI eruption can also explain the good fit reported in Costa *et al.* (2012), in which the effect of the gravity current in the umbrella cloud was not considered.

From a climatic perspective, recent studies indicate that the stratospheric aerosol cloud generated from the CI event would have induced a “volcanic winter”^25^ with a cooling effect of ~6–9 °C in Eastern Europe^26^. Using the total magma volume reported in this study (Table 2), and the CI melt composition^40^, we estimate (after Self *et al.* 2004) the amount of sulphur dioxide (SO_2_) released by the eruption to be 168–178 Tg of SO_2_ (84-89 Tg S), most of which reached the stratosphere, assuming negligible release in the troposphere^41^. Table 3 summarizes the volatile release estimates for each phase of the CI eruption. Our estimates are consistent with sulphate deposition records of the GISP2 ice core^42^. These values represent a 10–15% decrease compared to previous CI reconstructions as a single-phase event^3^ and are three times higher than those estimated for the largest historic eruption, the 1815 Tambora event^41^. Estimates of chlorine (Cl) and fluorine (F) are also calculated using the same methodology, taking into account the difference between concentrations dissolved in melt inclusions and those in matrix glass^43^. The amount of chemicals leached into the soil are calculated using volume estimations for the proximal pyroclastic density current deposits^21^. For large volcanic eruptions like the CI, stratospheric injection of SO_2_ is the principal atmospheric and global impact. In the stratosphere, SO_2_ is converted to sulphuric acid (H_2_SO_4_), which condenses rapidly to form fine sulphate aerosols that block incoming solar radiation and contribute to ozone destruction. Assuming a conversion efficiency (SO_2_ to sulphate aerosols) of ~86%^41^,^44^, the CI eruption would have yielded a maximum of 336–356 Tg of sulphate aerosols. These results are an order of magnitude greater than those found for the Mount Pinatubo eruption^44^, and are comparable with those of the Bishop Tuff eruption^45^.

The environmental stress that followed the CI eruption, aggravated by the onset of the Heinrich Event 4, provides a link between this exceptional volcanic eruption and the comprehensively discussed Middle to Upper Palaeolithic transition^28^,^46^,^47^,^48^. Despite a long history of investigation, considerable debate still focuses on whether Neanderthals became extinct as a result of climate change or due to competition with anatomically modern humans^22^,^49^. According to recent studies, a stratospheric aerosol cloud of the size indicated by our CI simulations would have induced a cooling effect of ~6–9 °C in eastern Europe and Northern Asia, 2–4 °C in Western Europe^26^, and ~1–2 °C globally^50^, with an e-folding decay time of approximately 1 year^51^. However, this “volcanic winter”^25^ would not have been sufficient to trigger dramatic changes in Upper Palaeolithic European populations on a larger scale^26^. Archaeological records indicate that anatomically modern humans from Central Asia and the Middle East first populated the European continent prior to the CI eruption, suggesting contemporaneity with Neanderthals^22^. Tephra fallout from the eruption would have reduced the area available for human settlement in Europe by up to 30% (Fig. 5), causing a halt in the westward dispersal of modern human groups and leading to a significant “genetic bottleneck”^28^. However, the removal of a large part of this tephra by erosion, the short acid deposition phase (1–2 years)^26^, and the availability of nutrient reserves in buried topsoils, would have allowed for a rapid (~10 years) ecosystem recovery^52^ in most areas away from the source. For example, the effect on net primary productivity following deposition of 5–10 cm of tephra from the 1980 eruption of Mount St. Helens was similar to that of subsequent yearly weather fluctuations^53^. This being considered, it is possible that modern humans would have gravitated towards repopulating these recovered areas rather than resuming their westward dispersal, permitting prolonged Neanderthal survival in South-Western Europe. This assumption is consistent with the existing consensus that Neanderthal populations persisted in southern Europe, particularly in southern Iberia, well after the CI eruption^28^. Furthermore, climatic changes from the Heinrich event 4 briefly created a biogeographic barrier between the Neanderthals and modern humans as described in the “Ebro Frontier” model^54^. Demographic pressure over this frontier after the reinstatement of modern human groups in central and Eastern Europe would have culminated in the assimilation of the last Neanderthal refugia through expansion from across the Pyrenees.

Finally, it is important to stress the model limitations associated to inverting tephra fallout (e.g. Connor & Connor, 2006)^29^. This kind of inversion bears a certain degree of inherent non-uniqueness, i.e., a variety of different ESPs combinations exist that can reproduce the deposit. In addition, a limited amount of sample deposits showing distinct phases of the eruption were available to reconstruct the CI event. However, despite the limitations of both model and measurements, we can consider our optimal ESPs as a model solution consistent with a statistical number of observations. The robustness of our solution is also corroborated from the fact that independent single/two-phase approaches give very similar results in terms of total volumes and durations as well as phase-averaged MER, column height, and mass distributions within the column.

## Methods

We applied a novel computation approach to infer ESP values for each phase of the CI eruption accounting for the gravitational spreading of the umbrella cloud. Our methodology uses the FALL3D tephra dispersion model in conjunction with a downhill simplex inversion method that selects a solution that best represents each phase of the eruption. Two independent datasets containing deposit thickness were used for inversion and validation. Finally, we calculated the amount of volatiles released for each phase of the eruption by using the volcanic emission estimate described in Self *et al.* (2004).

### Wind profiles

Costa *et al.* (2012) performed a five-step computational procedure to generate a set of five hundred synoptic meteorological fields (using 15 years of European Centre for Medium-Range Weather Forecasts (ECMWF) ERA-40 reanalysis data—see supplementary material in Costa *et al.* 2012) and concluded that the set of wind fields from 5–12^th^ of December 1991 was statistically representative of the (unknown) meteorological conditions at the time of the 39 ka CI eruption. We updated their methodology by using the ECMWF ERA-Interim reanalysis dataset at 0.25° × 0.25° resolution in the horizontal and 60 vertical levels (1 km) from the surface up to 0.1 hPa. Meteorological fields from the ERA-Interim dataset were interpolated over the FALL3D computational mesh with a 1-hour interval. The FALL3D mesh contained 241 × 201 × 50 nodal points. To improve the quality of the fit for the ultra-distal deposits, the wind field was rotated 7 degrees anti-clockwise around the vent. We found the wind fields from the 7^th^ of December 1991 to statistically best represent those at the time of the eruption.

### Geological datasets

#### Dataset 1

We used dataset 1 for the two-phase inversion. The dataset was derived from analysis of more than 40 marine, lacustrine and land deposits from across the dispersal area, originally presented in Engwell *et al.* (2014). The deposits range from 130 km from the source, at Lago Grande di Monticchio (LGdM), to distances of more than 2000 km, in Russia. Amongst these deposits, only the ten showing distinct bimodality were selected (Table 1). Within lake sediments at LGdM, the CI deposit was separated into a 16.5 cm thick coarse lapilli pumice fall overlain by a 13 cm thick vitric ash layer. This overlying ash layer, the co-ignimbrite layer, is fine grained with a median diameter of approximately 50 microns, and is relatively homogenous, with little variation in median diameter and sorting coefficient within the deposit. While the lapilli deposit fines slightly towards the top of the deposit, the boundary with the overlying vitric ash layer is sharp. Similar trends have also been noted within deposits at greater distances from source^10^, with the boundary between the two phases becoming more difficult to distinguish with distance. Deposits at greater distances were too thin for the two phases to be identified stratigraphically.

Deposits were chemically treated to remove any biological components, and grain size analysis was conducted using a combination of sieve and laser diffraction analysis^13^ using the Malvern Mastersizer 2000E. Deposits at LGdM are easily separated into Plinian and co-ignimbrite phases based on stratigraphy, with both deposits having a unimodal distribution. Grain size results for intermediate distances show the characteristic bimodality typical of deposits associated with multiple phases, and particularly those associated with Plinian and ignimbrite forming eruptions. Deposits at distances greater than 900 km from source are unimodal, and likely contain ash from both the Plinian and co-ignimbrite phase, however it was not possible to identify to what extent each component contributes^13^.

**Figure 2:**
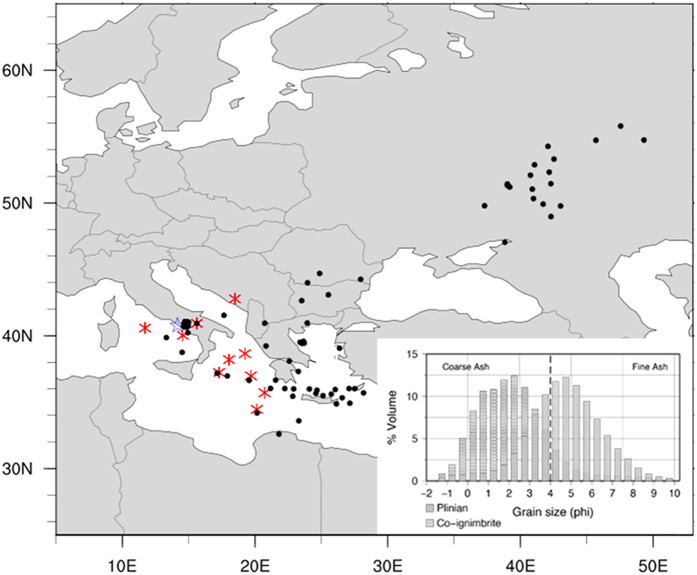
Map showing the location of the CI-caldera (star) and geological samples in dataset 1 (asterisks) and 2 (circles). The inset shows the reconstructed TGSD from dataset 1. The map was generated using the D3.js library.

**Figure 3:**
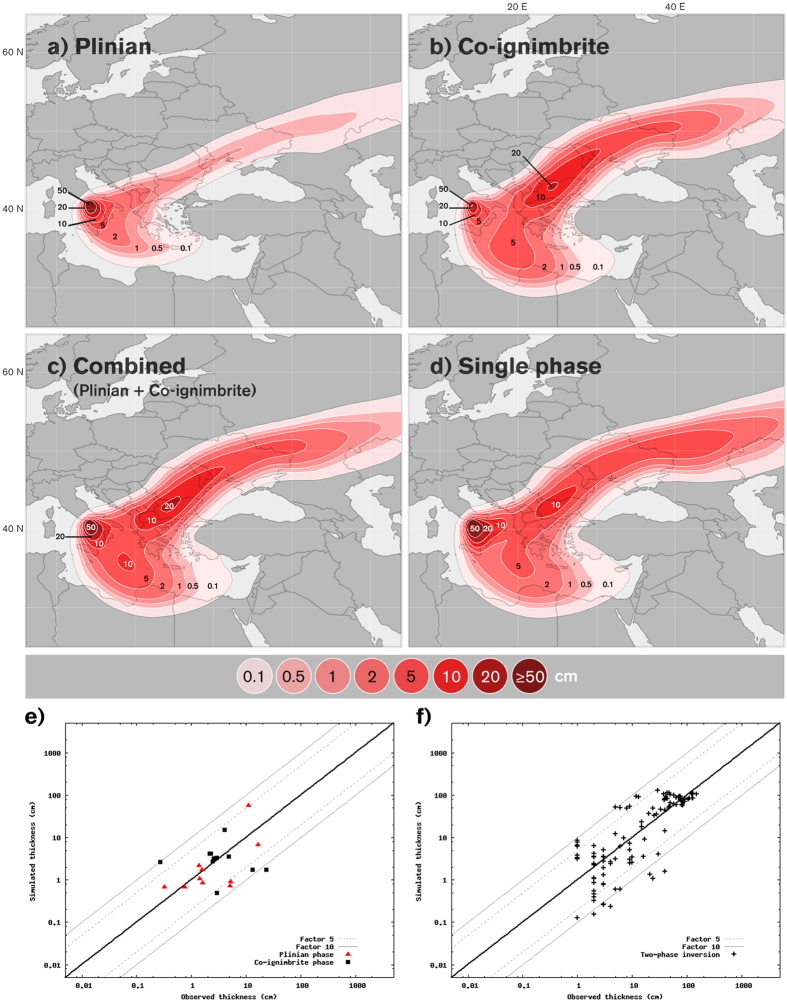
Isopach maps (cm) from inversion. (**a**) Plinian phase, (**b**) co-ignimbrite phase, (**c**) combined two-phase and, (**d**) single-phase. Bottom plots show simulated versus observed thicknesses for (**e**) Plinian and co-ignimbrite phases and (**f**) two-phase approach. The solid bold line represents a perfect agreement, while the dashed and solid thin black lines mark the region that is different from observed thicknesses by a factor 5 (1/5) and 10 (1/10), respectively. Topography data for map figures was obtained from Natural Earth. Figure generated using Autodesk® Maya® 2014.

**Figure 4:**
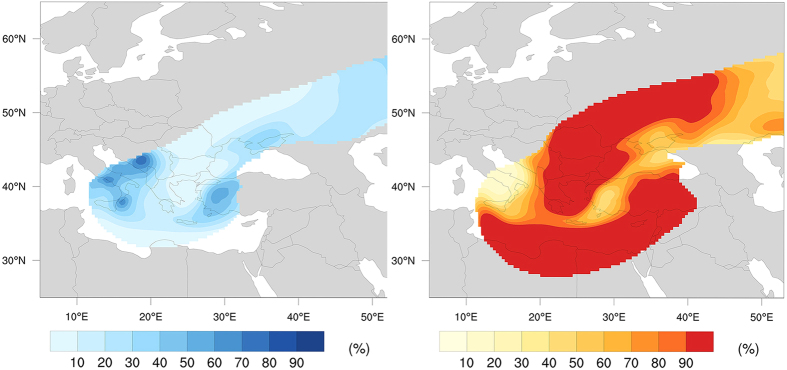
Contribution (%) from the Plinian (left) and co-ignimbrite (right) phases to the CI tephra deposit. Figure generated with NCAR Command Language (NCL).

**Figure 5:**
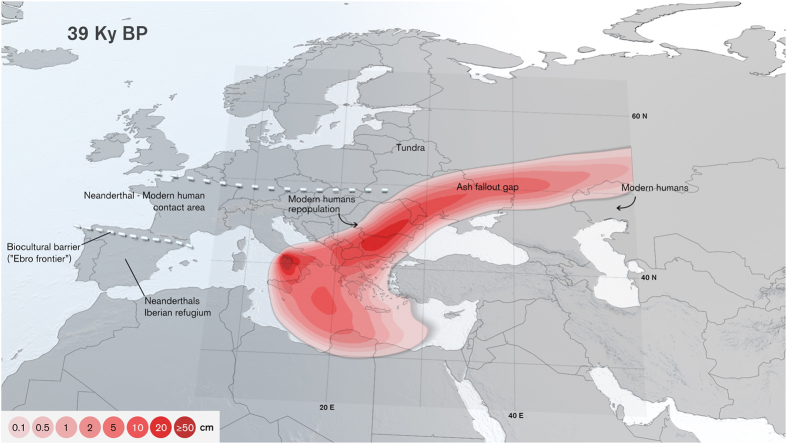
Campanian Ignimbrite’s contribution to the Middle to Upper Palaeolithic transition. Tephra fallout, together with the attendant episode of Fenno-Scandinavian ice cap and peripheral tundra advance on land (top dashed line), suggests a reduction of the area available for human settlement in Europe of up to 30% (represented by the ash fallout gap with isopach tephra deposits in cm). Anatomically modern humans would have gravitated towards repopulating this gap after ecosystem recovery, rather that overcoming new biogeographical frontiers, leading to an instance of prolonged Neanderthal survival in Iberian Peninsula. Topography data for map figures was obtained from Natural Earth. Figure generated using Autodesk® Maya® 2014.

We calculated the total grain size distribution of the two main component phases (inset Fig. 2) using the Voronoi Tessellation spatial statistical technique^55^, whereby the identified deposit extent is divided into a number of territories according to the spacing and distribution of the measurements. In this case, the deposit extent of Pyle *et al.* (2006) was used as the tessellation limit. Uncertainties associated with estimation of TGSDs are typically related to the choice of deposit extent and to the number and distribution of analysed deposits^55^,^56^. However, given the lack of variation in co-ignimbrite deposit grain size characteristics, regardless of direction and distance from source, the calculated TGSD is likely to be robust, and the resultant grain size is remarkably similar to co-ignimbrite deposits from other events (e.g. Montserrat^57^). In the case of the Plinian deposit, the fines from very distal deposits were not taken into account, and therefore the resultant grain size distribution is likely to underestimate the finest grain sizes.

#### Dataset 2

We used this dataset for the single-phase inversion and validation. The dataset consists of the 112 sample thicknesses previously used by Costa *et al.* (2012) plus two measurements in southeast Romania^34^. The online supplemental material S2 shows the location, thickness and distance from source of each geological sample.

### Tephra dispersal modelling

FALL3D is an Eulerian model for the transport and deposition of volcanic tephra. To determine the vertical distribution of mass within the plume through inversion we assumed an empirical parameterization^30^,^36^ that controls concentration of mass along the column. To account for aggregation processes, we used the empirical aggregation model of Cornell *et al.* (1983) proposed specifically for the CI eruption. For computational reasons, particle aggregation was assumed to occur within the eruption plume thereby affecting the original TGSD, which was modified considering a single aggregate class, depleting particle classes finer than the aggregate class itself. The horizontal diffusion coefficient was calculated using a large eddy parameterization such as the one used by the RAMS model^58^. The vertical diffusion coefficient was set to a constant value^59^ of 100 m^2^s^−1^. The particle settling velocity model of Ganser (1993) was used to predict settling rates of particles.

### Gravity current model

To account for the gravity-driven transport we coupled FALL3D with a parameterization that describes cloud spreading as a gravity current^16^. This model calculates an effective radial velocity of the umbrella spreading as a function of time, and combines it with the wind field centred above the vent in the umbrella region. To estimate the radial distance at which the critical transition between gravity-driven and passive transport occurs, we compared the umbrella front velocity with the mean wind velocity at the Neutral Buoyancy Level (NBL) estimating the Richardson number, Ri (gravity-driven regime when Ri > 1, passive transport regime for Ri < 0.25, and an intermediate regime in between these values).

### Inversion modelling and best-fitting criterion

The basic steps used in the inversion process for finding the optimal set of eruption parameters can be found in Connor & Connor (2006). We used a downhill simplex method (DSM) and the FALL3d dispersion model to infer the optimal values of the ESPs. Simulation results were compared with field measurements by employing a criterion as the goodness-of-fit measure test. ESP values were updated iteratively to minimize the difference between calculated values and observed measurements. Parameters were adjusted until the goodness-of-fit measure falls within the tolerance limit criterion. In addition to the minimization criterion used in Folch *et al.* (2010)^60^, we also considered the criterion use in Costa *et al.* (2014), originally proposed by Aida (1978) to measure the spatial variation between the recorded and computed tsunami heights^61^:


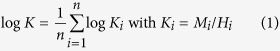



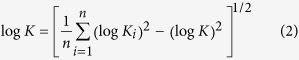


where *n* is the number of measurements, 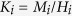 is the ratio of measured tephra thickness (load) to simulated thickness (load) at *i-th* location. The first Aida index (*K*) is associated with the geometric average of the distribution and the second (*k*) is related to the geometric standard deviation of the distribution. This approach was proven to be suitable for best-fitting tephra deposits^4^. As for tsunami simulations, we considered the simulated tephra thickness results satisfactory when


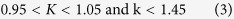


### Environmental and atmospheric emissions

To estimate the amount of volatiles released from the CI eruption, we used the volcanic emission estimate described in Self *et al.* (2004) for SO_2_, and updated these calculations to estimate fluoride and chloride emissions during the eruption:


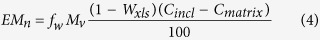


where *EM*_*n*_ is the emission for each substance (in kg), *M*_*V*_ is the mass of erupted magma (in kg) obtained from the our best-fit results, *W*_*xls*_ is the mass fraction of crystals in the magma^40^,^43^, (*C*_*incl*_ *−* *C*_*matrix*_) is the difference between the average chemical concentrations of the glass inclusions and the matrix in^24^ wt%, and *f*_*w*_ is factor difference between the molecular weights (e.g. factor 2 for the SO_2_ and S).

### Website

An interactive website providing a moderated explanation of this methodology and its results is available to the general public at: (http://www.bsc.es/viz/campanian_ignimbrite)

## Additional Information

**How to cite this article**: Marti, A. *et al.* Reconstructing the plinian and co-ignimbrite sources of large volcanic eruptions: A novel approach for the Campanian Ignimbrite. *Sci. Rep.* **6**, 21220; doi: 10.1038/srep21220 (2016).

## Supplementary Material

Supplementary Information

Supplementary Dataset 2

## Figures and Tables

**Figure 1:**
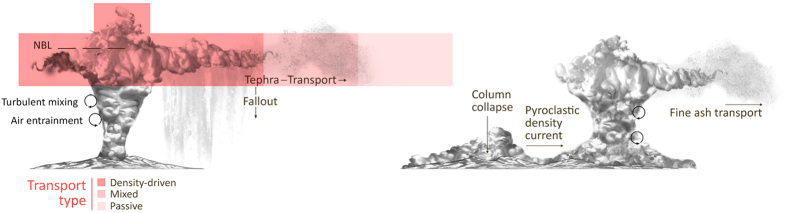
Schematic diagram (not to scale) of a super-eruption event with an initial (left) sustained Plinian phase followed by a column-collapse and large pyroclastic density currents eventually leading to co-ignimbrite plumes offset from the vent (right). Colour cells mark the extent of each transport regimes in the umbrella cloud.

**Table 1:** Dataset 1 showing tephra layer thicknesses from Engwell *et al.* (2014) for Plinian and co-ignimbrite phases of the CI eruption with distance from the source.

Sample	Lon (E)	Lat (N)	Depositional Environment	Water Depth (m)	Distance from source (km)	Thickness Plinian (cm)	Thickness Co-ignimbrite (cm)	% Co-ignimbrite Tephra
TR172-42	14.55	40.02	Deep Sea	728	118	11	4	27.7
LGdM	15.61	40.93	Lake	NA	130	16.5	13	44.1
MONTEN	18.48	42.78	Cave	2,750	430	5.1	2.9	36.2
RC9-191	18.03	38.20	Deep Sea	2,345	445	1.56	2.44	61.0
V10-69	17.28	37.23	Deep Sea	3,156	490	1.38	1.62	54.0
RC9-190	19.23	38.65	Deep Sea	1,712	497	1.4	2.6	65.0
RC9-189	19.68	36.98	Deep Sea	3,378	645	5.2	4.8	48.0
V10-67	20.72	35.70	Deep Sea	2,904	810	1.6	2.9	64.4
RC9-185	20.12	34.45	Deep Sea	2,858	890	0.75	2.25	75.0
TR171-21	20.13	34.45	Deep Sea	2,785	900	0.32	2.18	87.2

MONTEN values calculated from data in Morely & Woodward (2011)^62^. Note how the percentage of the co-ignimbrite contribution tends to increase with distance from source.

**Table 2:** Best-fit results obtained from reconstructing the CI super-eruption as a two-phase and single-phase event.

Modelled dispersion parameters	Explored Range	TWO-PHASE			SINGLE-PHASE
		Plinian phase	Co-ignimbrite phase	Combined phases	Single phase event
Tephra mass (kg)	Calculated	5.40 × 10^13^	1.54 × 10^14^	2.08 × 10^14^	2.11 × 10^14^
Average deposit density (kg/m^3^)(a)	Assumed	1,000	1,000	1,000	1,000
Tephra volume (km^3^)	Calculated	54	153.9	207.9	211.1
Tephra volume DRE (km^3^)	Calculated	22.6	61.6	84.2	84.4
Duration (h)	12–48	4	19	23	23
Mass eruption rate (kg/s)	10^8^–10^10^	3.75 × 10^9^	2.25 × 10^9^	2.51 × 10^9^(b)	2.55 × 10^9^
Column height (km)	20–50	44	37	38(b)	38
TGSD modes (Φ)(c)	0–3/6–9	2.5(d)	5(d)	—	2.0/6.5(c)
TGSD variances (Φ)(c)	1–3/1–3	1.16	1.22	—	2/2(c)
Suzuki coefficient A (−)(e)	2–9	4	9	8(b)	9
Density of aggregates (kg/m^3^)(f)	100–500	350	350	350	350
Diameter of aggregates (in Φ–unit)(f)	2–3	2.3	2.3	2.3	2.3
Pearson correlation coefficient (R)(h)	Calculated	0.76	0.83	0.81	0.79
Root-mean-square error (RMSE)(h)	Calculated	0.10	0.30	0.18	0.27
Aida indexes K/k (−)(i)	Calculated	1.01/1.05	1.03/1.07	1.02/1.14	1.02/1.15

The combined phase column is obtained by using the optimal ESPs resulting from the Plinian and co-ignimbrite phase inversions.

^(a)^This value is used to convert mass loading to deposit thickness and thereby to calculate tephra volume from mass, whereas a bulk density of 2500 kg/m^3^ was considered to convert into DRE volume.

^(b)^Weighted sum of input parameters for each phase.

^(c)^Total grain size distribution (TGSD) for the single phase reconstruction is assumed bi-Gaussian in Φ with maxima at μ_1_ and μ_2_ and corresponding variances σ_1_ and σ_2_.

^(d)^TGSD for the two-phase reconstruction was determined by Voronoi tessellation^55^.

^(e)^The eruption source is described in a purely empirical way using the Suzuki distribution^30^,^36^ for mass release along the column.

^(f)^Aggregation is accounted for using the empirical model of Cornell *et al.* (1983), assuming that 50% of the 63–44 μm ash, 75% of the 44–31 μm ash, and 95% of the less than 31 μm ash fell as aggregated particles, with diameter and density of aggregates found through the best-fit.

^(h)^Pearson correlation (R) and root-mean-square error (RMSE) based on the differences between log (measured thickness) and log (simulated thickness).

^(i)^Aida index for geometric average (*K*) and geometric standard deviation (*k*) of the distribution.

**Table 3:** Chemical release estimates by each phase of the CI eruption.

Chemical	Stratospheric volatiles (Tg)			Leached into the soil (Tg) (by proximal PDC)
	Plinian phase	Co-ignimbrite phase	Combined phases	
SO_2_ aerosols	88–92	248–264	336–356	n.a.
SO_2_	44–46	124–132	168–178	273–289
Fluoride	243–256	693–731	936–987	1,519–3,384
Chloride	340–359	970–1,024	1,310–1,383	2,362–4,738

Left: estimation of stratospheric volatiles after Self *et al.* (2004); Right: chemicals leached into the soil considering volume estimations for the proximal pyroclastic density current deposits after Pyle *et al.* (2006).
