# Supplementary material for: Reconstructing the plinian and co-ignimbrite sources of large volcanic eruptions: A novel approach for the Campanian Ignimbrite
Source: Sci Rep. 2016 Feb 17;6:21220. doi: 10.1038/srep21220 (PMC4756320; doi:10.1038/srep21220)
Supplement: Supplementary Information [file srep21220-s1.pdf]

SUPPLEMENTAL INFORMATION

Reconstructing the plinian and co-ignimbrite sources of large volcanic eruptions:

A novel approach for the Campanian Ignimbrite

Alejandro Marti[1,*], Arnau Folch[1], Antonio Costa[2], and Samantha Engwell[3]

[1] Barcelona Supercomputing Center (BSC-CNS), Barcelona, Spain
[2] Istituto Nazionale di Geofisica e Vulcanologia, Sezione di Bologna, Italy
[3] Istituto Nazionale di Geofisica e Vulcanologia, Sezione di Pisa, Italy
[*]Alejandro.Marti@bsc.es

SUPPLEMENTAL INFORMATION S1

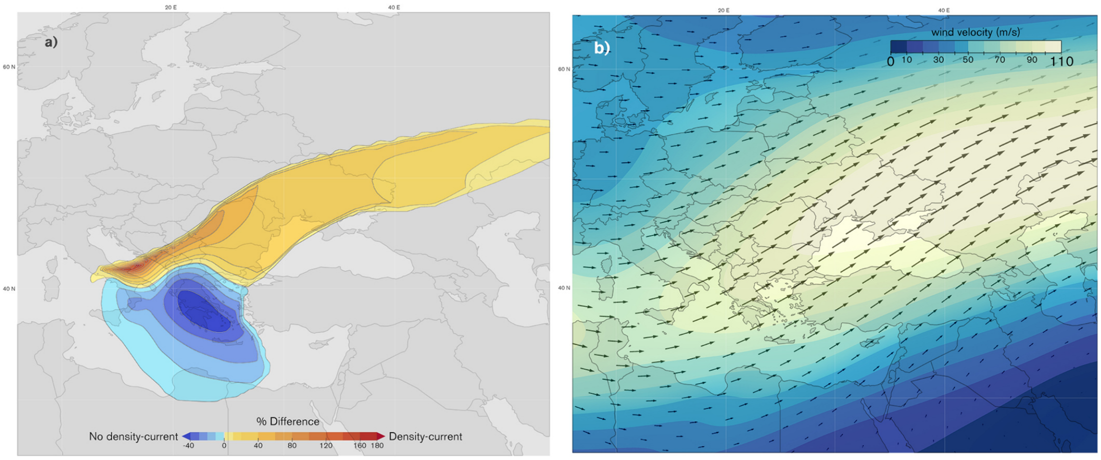

Figure S1. (a) Impact of gravity current on the deposit (variation in %). Positive values show areas where tephra deposition increases when accounting for gravity-current effects; negative indicate lesser tephra deposition. (b) Wind field at ~32 km elevation for the meteorological field that best represents the (unknown) meteorological conditions at the time of the CI eruption (7th December 1991). Figure generated using Autodesk(R) Maya(R).
